# Bilateral transversus abdominis plane (TAP) block reduces pain and the need for additional analgesics after elective cesarean section under opioid-free spinal anesthesia: findings from a randomized clinical trial

**DOI:** 10.1186/s44158-023-00106-6

**Published:** 2023-06-26

**Authors:** Massimo Antonio Innamorato, Alessandro Vittori, Silvia Natoli, Paolo Perna, Ilaria Farinelli, Emiliano Petrucci, Marco Baciarello, Elisa Francia, Franco Marinangeli, Elena Giovanna Bignami, Marco Cascella

**Affiliations:** 1 Department of Neuroscience, Santa Maria Delle Croci Hospital, AUSL Romagna, Pain Unit, 48121 Ravenna, Italy; 2 Department of Anesthesia and Critical Care, ARCO Roma, Ospedale Pediatrico Bambino Gesù IRCCS, 00165 Rome, Italy; 3 Department of Clinical Science and Translational Medicine, University of Rome Tor Vergata, 00133 Rome, Italy; 4 IRCCS Maugeri Pavia, 27100 Pavia, Italy; 5 Department of Anesthesia and Critical Care, Ospedale Maggiore Carlo Alberto Pizzardi, 40133 Bologna, Italy; 6 Department of Anesthesia and Intensive Care Unit, San Salvatore Academic Hospital of L’Aquila, 67100 L’Aquila, Italy; 7 Critical Care and Pain Medicine Division, Department of Medicine and Surgery, University of Parma, 43126 Parma, Italy; 8 Department of Anesthesiology, Intensive Care and Pain Treatment, University of L’Aquila, 67100 L’Aquila, Italy; 9 Department of Anesthesia and Critical Care, Istituto Nazionale Tumori-IRCCS, Fondazione Pascale, 80131 Naples, Italy

**Keywords:** Cesarean section, Bilateral transversus abdominis plane (TAP) block, Postoperative pain, Opioid-free anesthesia, Pain, Non-steroidal anti-inflammatory drugs, Anesthesia, Neonate, Locoregional anesthesia, Analgesia

## Abstract

**Background:**

Cesarean section (CS) is the most frequently performed obstetric procedure globally, and postoperative pain remains a prominent concern. This study aimed to evaluate the effectiveness of the bilateral transversus abdominis plane (TAP) block in addressing this issue.

**Methods:**

We performed a randomized trial in women with term pregnancies who underwent elective CS with spinal anesthesia. The women were randomized (1:1) to receive bilateral TAP or postoperative systemic analgesics (control group). The primary outcome was the effect on postoperative pain assessed using the numeric rating score (NRS) at 2, 6, 12, and 24 h in the postoperative period.

**Results:**

At 2 and 6 h after the surgical procedure, there was a significant reduction in both resting (rNRS *p* = 0.004) and movement-related pain (dNRS *p* = 0.0001, *p* = 0.001 respectively). However, at 12 h, a reduction of dNRS was demonstrated (*p* = 0.0001), while no benefit was observed at rest. The percentage of women with NRS ≤ 4 was higher after the block at 2 h for both resting and movement-related pain (rNRS *p* = 0.010; dNRS *p* = 0.0001); at 6 and 12 h, it was only significant for dNRS (*p* = 0.002). Rescue doses of analgesics were significantly higher in the control group at 2, 6, and 12 h (*p* = 0.01, *p* = 0.0383, *p* = 0.0003 respectively). No complications with the procedure were recorded.

**Conclusion:**

Bilateral TAP block has the potential to alleviate postoperative pain and reduce the need for additional analgesics after CS.

**Trial registration:**

This study is registered with ClinicalTrials.gov, number (NCT02801968), registered 28 May 2016, https://clinicaltrials.gov/ct2/show/NCT02801968?term=NCT02801968&draw=2&rank=1

## Background

Cesarean section (CS) is a widely practiced surgical procedure worldwide, with approximately 20% of women currently undergoing this method of delivery [1]. Moreover, since the incidence of CS is increasing in recent decades [2], it is important to define optimal strategies for perioperative management.

One crucial aspect is the management of postoperative pain as moderate-to-severe pain is reported in up to 80% of women [3]. This represents a significant challenge given that providing women who undergo a CS with optimal postoperative analgesia is crucial for effective pain control and has wide-ranging positive impacts. Effective pain relief, for example, improves mobility, facilitating recovery and engagement in daily activities [4]. Furthermore, a fruitful pain management strategy also promotes successful breastfeeding, contributing to the health and well-being of both the mother and the neonate. Additionally, adequate pain control enhances the maternal bond with the neonate, enabling mothers to engage in crucial activities such as skin-to-skin contact, holding and caring for their infants, and establishing a deep emotional connection. Moreover, inadequate pain control could also lead to the development of chronic pain in approximately 12% of patients [5] and trigger postpartum depressive syndromes [6]. Therefore, optimal pain management supports the development of a strong and loving relationship between mother and neonate and can prevent the occurrence of postoperative complications.

There are several approaches utilized to enhance post-cesarean analgesia and ensure adequate pain management. These include the systemic administration of opioids, the use of non-steroidal anti-inflammatory drugs (NSAIDs), regional anesthetic techniques (spinal with low-dose intrathecal morphine or other opioids, and epidural anesthesia), regional nerve blocks, wound infiltration or continuous wound infusion, and the implementation of patient-controlled analgesia [7]. Multimodal analgesic approaches involve combining different medications and techniques to target pain from various angles. Consequently, synergistic pain relief can be achieved with lower doses and reduced side effects [8].

Following a CS, women may experience both somatic pain, originating from the abdominal wall incision, and visceral pain caused by the uterus. The predominant source of discomfort is often the abdominal wall, which is of somatic origin. Transversus abdominis plane (TAP) block is a regional anesthesia technique that involves injecting a local anesthetic into the transversus abdominis (TA) plane. It is a triangular fascial plane located between the internal oblique (IO) and the TA muscles. The interstitial space mentioned encompasses the intercostal, subcostal, iliohypogastric, and ilioinguinal nerves. Since these nerves innervate the anterior and lateral abdominal wall as well as the parietal peritoneum, this block can provide targeted pain relief to the incision site and surrounding areas, reducing the need for systemic analgesics and improving postoperative comfort [9].

We performed a prospective, randomized trial comparing postoperative pain relief after TAP block versus systemic analgesia in patients undergoing elective CS with opioid-free spinal anesthesia. We hypothesized that TAP block would provide better pain control compared with systemic administration of opioids and non-opioid agents.

## Materials and methods

The study was approved by the Research Ethics Committee of the institution (Ethical Committee Area Vasta Romagna), opinion 1574, on April 13, 2016 and it was registered with ClinicalTrials.gov, number (NCT02801968). It was carried on in accordance with the Helsinki Declaration. This investigation adheres to the Consolidated Standards of Reporting Trials (CONSORT) guidelines [10].

### Trial design and procedures

From June 1, 2016, through June 30, 2016, we conducted a single-center randomized clinical trial at the Obstetric Anesthesia Unit, Santa Maria Hospital, Ravenna, Italy. Enrollment for the study included women who were at least 18 years old and had term pregnancies with a gestational age ranging from 37 to 42 weeks. These women were scheduled to undergo elective CS under spinal anesthesia. Details of the inclusion and exclusion criteria can be found in Table 1.

**Table 1:** Inclusion and exclusion criteria

*Type*	*Criteria*
*Inclusion*	Women 18 years of age and older
	Term pregnancies (37–42 weeks) and scheduled for elective cesarean delivery under spinal anesthesia
	Able to provide informed consent, comply with the study visit schedule, and successfully complete all study assessments
	Positive recommendation for performing the TAP block during the anesthetic consultation
*Exclusion*	BMI > 35 kg/m^2^ or anatomic conditions that may preclude the regional block
	Increased susceptibility to bleeding or a coagulation disorder (a platelet count below 80,000 × 10^3/mm^3 or an INR > 1.5)
	Any known allergy, hypersensitivity, intolerance, or contraindication to any of the study medications, such as local anesthetics, opioids, or NSAIDs
	Relevant clinical conditions in either the mother or the neonate, such as gestational hypertension, impaired renal or hepatic function, post-partum hemorrhage requiring treatment, and others at the discretion of the investigators
	Chronic pain conditions and concurrent analgesics use
	Documented history, suspicion, or known addiction or abuse of illicit drugs, prescription medications, or alcohol (previous 2 years)

*Abbreviations*: *TAP* transversus abdominis plane, *BMI* body mass index, *INR* international normalized ratio, *NSAIDs* nonsteroidal anti-inflammatory drugs

All women then received intrathecal hyperbaric bupivacaine hydrochloride 12.5 mg (0.5%). After the end of the surgery, eligible women were randomized in a blinded 1:1 ratio and assigned to a treatment group:

#### Group A

Bilateral TAP block for postoperative analgesia. The TAP block was performed under ultrasound (US) guidance with a 6–15 MHz linear transducer. Patients were scanned in the supine position between the inferior margin of the 12th rib and the iliac crest, along the midaxillary line to find the best visualization of the obliquus externus, OI, and TA muscles. A 150-mm 20 G needle (Ultraplex ®, B. Braun Milano, Milan, Italy) was used to inject 0.5% ropivacaine (20 mL on each side). Postoperative analgesia was provided with tramadol (100 mg IV) q8h and acetaminophen 1000 mg q8h. Rescue doses with analgesics were allowed (ketorolac 30 mg, max 90 mg/day).

#### Group B

Standard systemic intravenous postoperinative analgesia with IV tramadol 100 mg q8h, acetaminophen 1000 mg q8h, and ketorolac 30 mg prn q8h.

### Endpoints

The primary endpoint of the study focused on evaluating the impact of the intervention on postoperative pain levels. Pain intensity was assessed using the numeric rating score (NRS) at specific time points within the postoperative period, including 2, 6, 12, and 24 h. We recorded pain intensity both at rest (rNRS) and during voluntary contraction of the abdominal muscles (dynamic, dNRS). Furthermore, the percentage of women with an NRS score of 4 or lower was also considered.

The secondary objective of the study was to assess the postoperative consumption of the nonsteroidal anti-inflammatory drug (NSAID) ketoprofen, patient satisfaction, and the occurrence of block-related side effects including local anesthetic systemic toxicity due to intravascular injection, neurologic injury (e.g., nerve trauma from the needle), visceral trauma, and vascular injury.

Patients’ overall satisfaction was registered 24 h postoperatively. Participants were asked to assess their level of satisfaction based on their experience by using a three-tier scale including “very satisfied,” indicating a high degree of contentment; “satisfied,” indicating a general sense of satisfaction; and “dissatisfied,” indicating a lack of satisfaction or dissatisfaction.

### Sample size calculation and statistical analysis

The null hypothesis of the study postulated that there would be no significant reduction in pain intensity 12 h after the surgical procedure. In order to ascertain a ~ 70% difference in intensity with a risk of type I error ≤ 5% and of type II error ≤ 20%, we calculated that a sample size of 31 per group was necessary. Data were reported as median (interquartile range, IQR) or number (percentage of the group). Group results were compared with *t* tests, Mann–Whitney, and *χ*2 tests, according to data distribution. Data manipulation and analysis were done in Microsoft Excel, SPSS Statistics 20, and MedCalc.

## Results

**Fig. 1:**
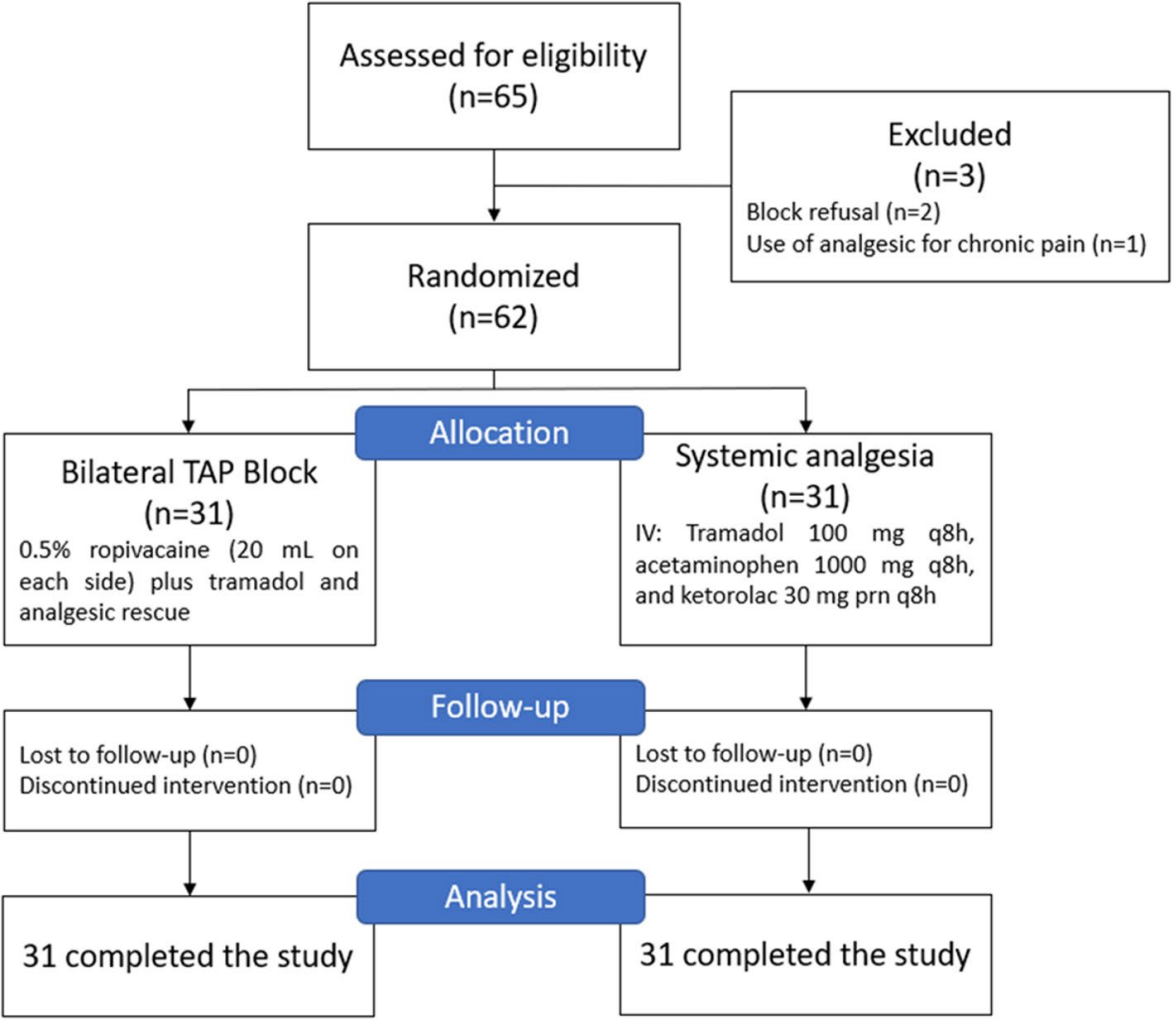
CONSORT flow diagram

A total of 65 women scheduled for CS were screened for eligibility. Of these, 62 met the criteria for inclusion in the study. Three women were excluded due to refusal of the regional analgesic technique (*n* = 2) and due to chronic use of analgesics (*n* = 1). After allocation, all the women completed the study (Fig. 1).

There were no significant differences in baseline characteristics between the two groups (Table 2).

**Table 2:** Baseline characteristics

	*TAP block (n* = *31)*	*Systemic analgesia (n* = *31)*	*P* value
*Age (y*ears*), median, SD*	33.87(4.87)	34.54 (5.05)	0.613
*Weight pre-pregnancy (kg), median, SD*	61.87(7.81)	60.16(7.81)	0.317
*Weight at delivery (kg), median, SD*	72.64(7.14)	71.74(6.51)	0.522
*Body* mass index *(kg/m*^*2*^*)*	24.85(1.96)	26.58(1.83)	0.090
*Gestational age (days)*	272.3(4.86)	272.9(5.12)	0.640
*Surgical duration (min)*	53(8)	52(8)	0.47
*American Society of Anesthesiologists status*	2(1–2)	2(1–2)	0.5
*Previous C-section (%)*	14 (46%)	12 (39%)	0.606

### Primary endpoint

#### Postoperative resting and dynamic pain

Two hours after the completion of the surgical procedure, compared to the control group, the TAP block group reported a statistically significant reduction in NRS values both at rest (*p* = 0.004) and during movement (*p* = 0.0001).

In 6 h, a statistically significant reduction in NRS values was observed both at rest (*p* = 0.001) and during movement (*p* = 0.004).

After 12 h, the mean NRS value at rest did not show significant differences between the two groups (*p* = 0.058); however, the mean NRS value during movement (dNRS) was significantly lower in the TAP block group (*p* = 0.0001).

At 24 h, no significant difference in pain was observed between the two groups, both at rest (*p* = 0.067) and during movement (*p* = 0.218) (Table 3).

**Table 3:** Resting numeric rating scale (rNRS) and dynamic numeric rating scale (dNRS) values at different time intervals

	**TAP block**	**Control group**	***P* value**
rNRS 2 h	3(2–3)	3(3–4)	0.004
dNRS 2 h	4(3–4)	4(4–5)	0.0001
rNRS 6 h	3(2–4)	4(3–4)	0.004
dNRS 6 h	4(3–5)	5(5–6)	0.001
rNRS 12 h	3(2–4)	4(3–4)	0.058
dNRS 12 h	5(4–5)	6(5–6)	0.0001
rNRS 24 h	2(2–3)	3(2–3)	0.067
dNRS 24 h	4 (3–5)	4(4–5)	0.218

Data are presented as mean and interquartile ranges

#### Pain control in the two groups (NRS ≤ 4)

**Fig. 2:**
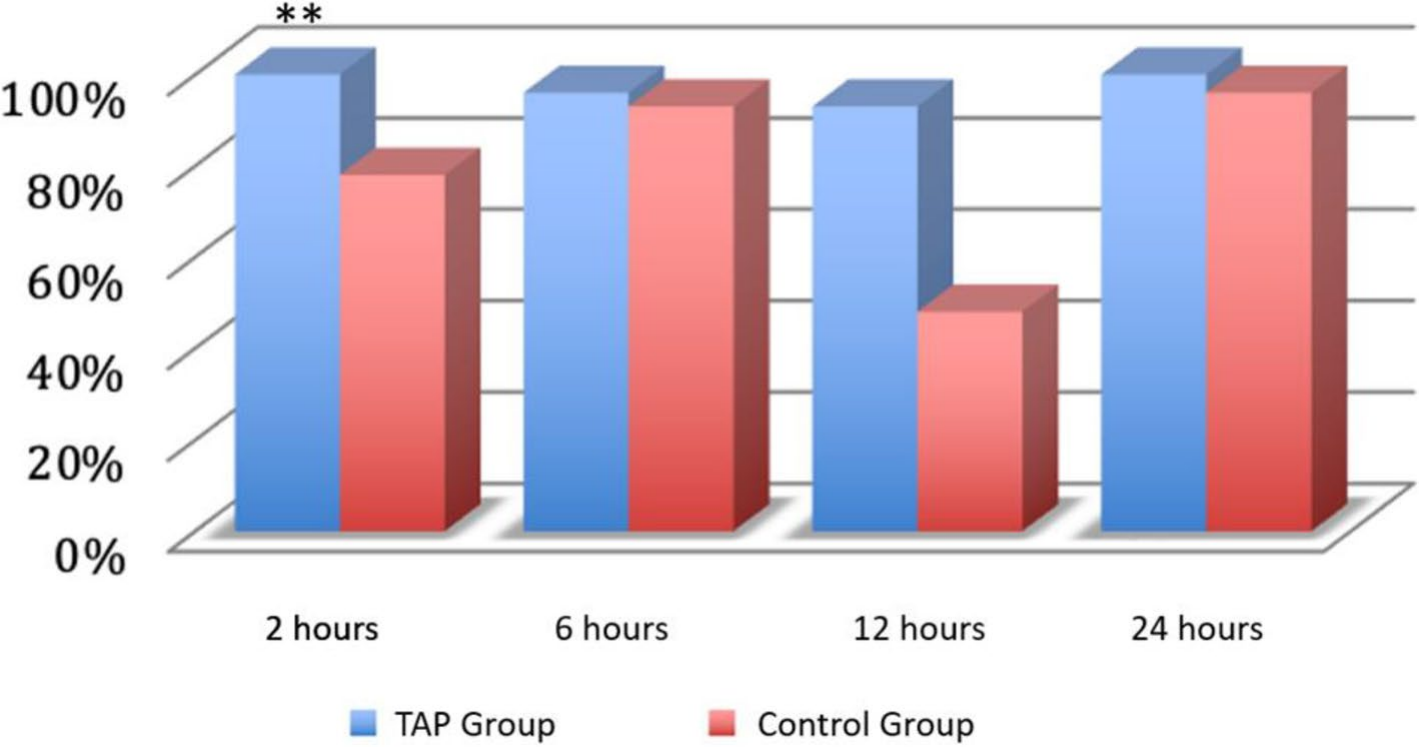
Pain at rest. Legend: A value below 4 on the NRS scale was considered. On the *y* axis, the percentage of patients. *p < 0.05; **p < 0.01; ***p < 0.001; ****p < 0.0001

Following the end of the intervention, 2 h later, 95% of women in the TAP block group reported an NRS value ≤ 4 at rest compared to 78% in the control group (*p* = 0.010) (Fig. 2).

**Fig. 3:**
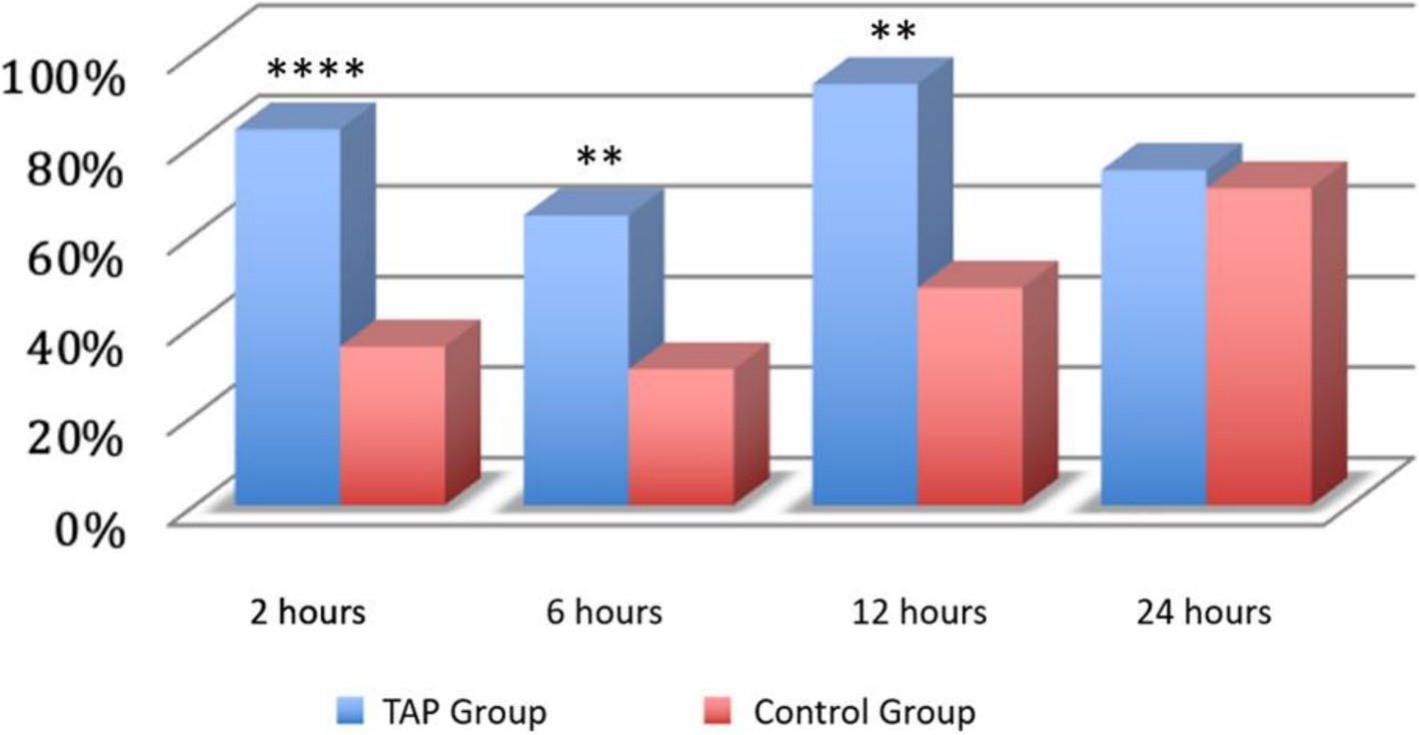
Pain during voluntary contraction of the abdominal muscles. Legend: A value below 4 on the NRS scale was considered. On the y-axis, the percentage of patients. *p < 0.05; **p < 0.01; ***p < 0.001; ****p < 0.0001

During movement, 79% of the patients in the TAP block group had an NRS ≤ 4 compared to 35% in the control group (*p* = 0.0001) (Fig. 3).

At 6 h, the proportion of patients with an NRS ≤ 4 at rest at 6 h was very similar in the two groups, 96% and 93% respectively (*p* = 0.553) (Fig. 2), while during movement, the TAP block group had a proportion of patients with an NRS ≤ 4 of 64% compared to 30% in the control group, with a statistically significant difference (*p* = 0.002) (Fig. 3).

After 12 h, all the patients in the TAP block group (100%) reported an NRS ≤ 4 at rest 12 h after the surgical procedure compared to 93% in the control group (*p* = 0.154) (Fig. 2). During movement, 48% of the patients in the TAP block group had an NRS ≤ 4 compared to only 30% in the control group (*p* = 0.002) (Fig. 3).

After 24 h, all the patients in the TAP block group reported an NRS ≤ 4 at rest 24 h after the surgical procedure compared to 96% in the control group (*p* = 0.313). During movement, 74% of the patients in the TAP block group had an NRS ≤ 4 compared to 70% in the control group (*p* = 0.775) (Fig. 3).

### Secondary endpoint

**Fig. 4:**
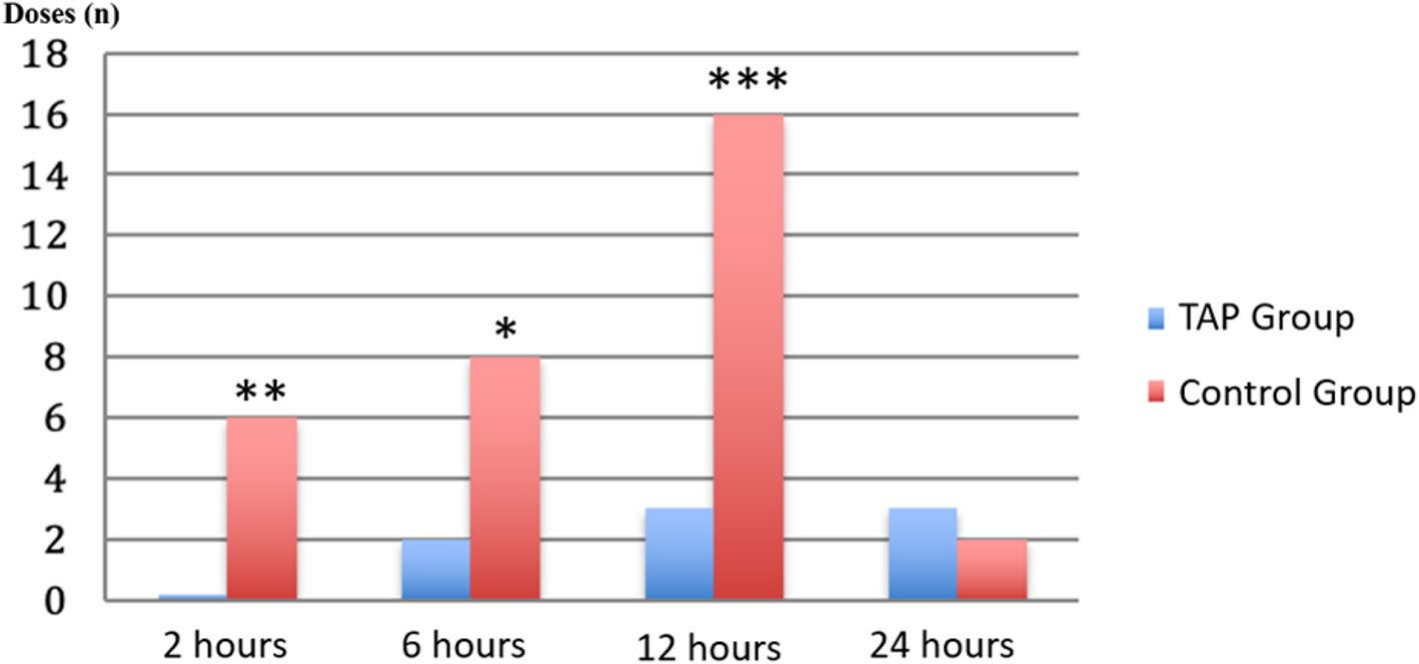
Postoperative rescue doses of ketoprofen. Legend: *p < 0.05; **p < 0.01; ***p < 0.001; ****p < 0.0001

**Fig. 5:**
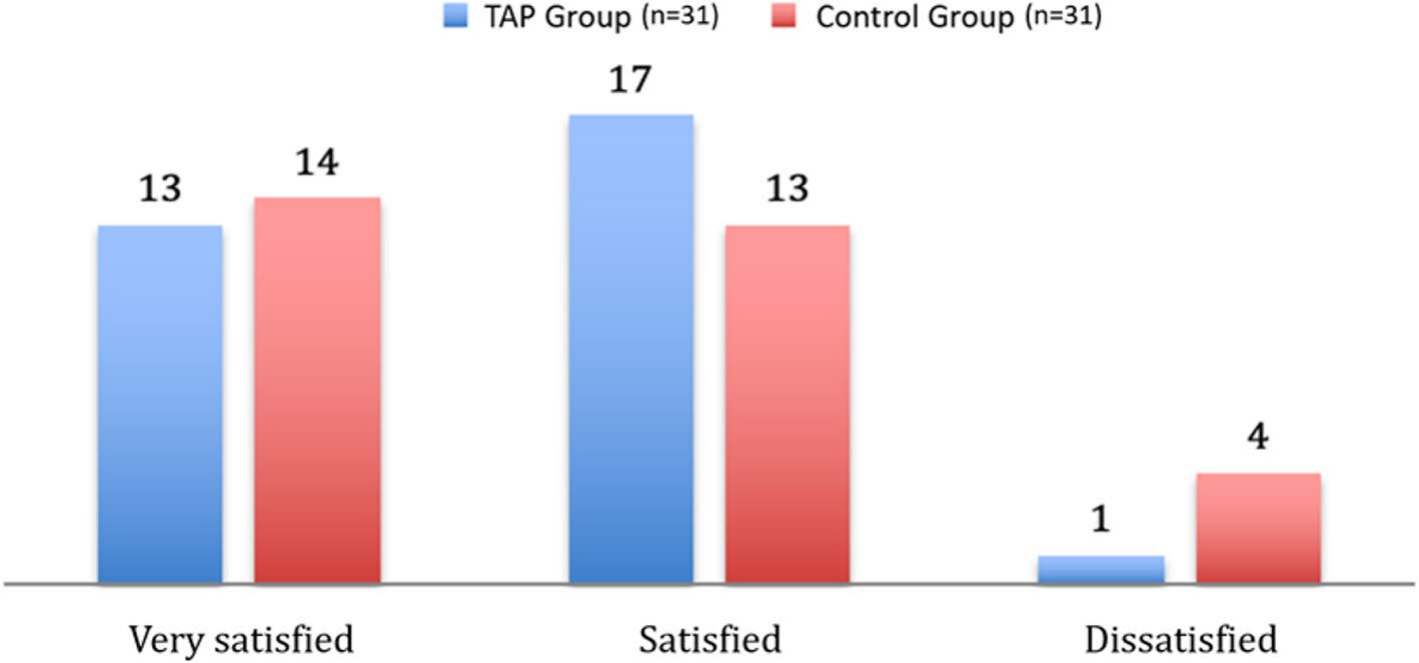
Patients’ overall satisfaction registered 24 h postoperatively

The use of NSAIDs differed significantly between the two groups after the administration of the block at 2, 6, and 12 h. In the TAP block group, no patients required additional analgesia 2 h after the surgical procedure, while in the control group, NSAIDs were administered to a total of 6 patients (*p* = 0.01). At 6 h, the consumption of ketoprofen differed significantly between the two groups (*p* = 0.0383), as well as at 12 h (*p* = 0.0003). There was no difference between the two groups at 24 h (*p* = 0.640) (Figs. 4 and 5).

There were no complications during the performance of the TAP blocks.

## Discussion

Bilateral US-guided TAP block combined with opioid-free spinal anesthesia, as compared with a regimen of systemic opioids and no-opioid analgesics, mitigated the intensity of postoperative pain following CS. At 2 and 6 h after the surgical procedure, there was a significant reduction in both resting and movement-related pain. However, at 12 h a reduction in pain during movement was demonstrated, while no benefit was observed at rest. The proportion of women experiencing NRS scores of 4 or lower was higher in the block group at 2 h for both resting pain and pain during movement. At 6 and 12 h, a significant difference was observed only for pain during movement. These findings strongly confirm results from evidence-based analyses [11, 12] and suggest further investigations to refine the technique, molecules, and dosages to be used.

The TAP block is an effective and safe locoregional anesthesia technique used in numerous surgical procedures with excellent results [13]. Several studies have shown that this strategy may reduce pain after CS in the context of multimodal analgesia including intrathecal or systemic morphine and other opioids [6, 14]. However, in our protocol, the intraoperative administration of agents belonging to this class of drugs was not planned. This data is of utmost importance considering the potential short-term and long-term side effects attributed to perioperative opioids [15–17]. For example, while low-dose intrathecal morphine is widely regarded as the preferred method for postoperative pain management following a CS section performed under spinal anesthesia, it carries the risk of undesired opioid-related side effects including pruritus, nausea, vomiting, urinary retention, and sedation [18].

During the initial hours after the cesarean section, the implementation of the TAP block procedure exhibited a notable improvement in analgesic efficacy, effectively alleviating both resting and movement-related pain experienced by the mothers. This substantial pain relief provided a considerable advantage, contributing to overall comfort and well-being. However, it is particularly noteworthy that the most significant impact of the procedure was observed at the 12-h mark, specifically in relation to pain experienced during movement. This finding carries paramount importance as it underscores the potential of the TAP block technique to facilitate and enhance maternal activities, particularly during critical moments of mother-infant integration. By effectively reducing pain during movement, the TAP block can offer mothers a greater degree of mobility and physical comfort, enabling them to actively engage in essential tasks such as breastfeeding and caring for their newborns. These activities, which are crucial for establishing a strong maternal bond, are often hindered by postoperative pain [4].

In our study, the superiority of TAP over the control group decreased at the 12 h and the 24th hour. However, the assessment of the pain indices reveals that both enrollment groups achieved satisfactory levels of pain control (Table 3). This highlights the effectiveness of a multimodal approach. In fact, during these intervals, there were fewer rescue doses administered (Fig. 4) and a higher percentage of patients reported NRS < 4 (Figs. 2 and 3). This data is noteworthy because, according to several studies, intrathecal morphine provides superior analgesia compared to TAP blocks up to 24 h after CS [19, 20].

Despite the use of ropivacaine has likely resulted in a longer duration of analgesic effect, the decrease in the usage of supplementary analgesic medications is significant in the TAP group at 2, 6, and 12 h, after which the block no longer ensures pharmacological savings. Other authors have employed plain bupivacaine; however, it has a short duration of analgesia (approximately 5–8 h) [6]. In other experiences, liposomal bupivacaine has been utilized [21]. This is another viable alternative to increase the duration of the block; however, this drug is not always readily available, and its use does not appear to be associated with cost reduction [22].

In contrast to the findings of previous studies [23], which reported improvements in perceived quality among patients, our research did not observe a similar outcome. This discrepancy could potentially be attributed to the methodology employed in our study. It is possible that the use of a more comprehensive and validated tool, such as a detailed questionnaire or a Likert scale, would have provided a more accurate assessment of patients’ perceptions.

## Study limitations

This study is limited to a single center, meaning that the findings may be influenced by specific characteristics and practices unique to that particular institution. While efforts were made to include a diverse study population, the generalizability of the results may be influenced by this limitation. Nevertheless, the procedure was performed by experienced anesthesiologists using a standardized technique.

This study acknowledges a limitation in the lack of data regarding general postoperative complications such as nausea and vomiting, and pruritus. In a recent investigation, these complications were most frequent in the study group of TAP plus spinal anesthesia, compared to general anesthesia, and peridural anesthesia, combined or not with the regional block [6]. On the other hand, the correlation between the decrease in opioid usage and a reduction in opioid-related side effects is not consistently supported by the evidence and remains inconclusive [19]. To address this gap, future research should consider incorporating comprehensive assessment and reporting of these complications. This could involve implementing standardized protocols for monitoring and documenting postoperative symptoms. Additionally, collecting patient-reported outcomes and conducting follow-up assessments can provide valuable insights into the occurrence and impact of these complications.

One limitation of our study is the reliance on opioids (tramadol) for postoperative pain control. However, it is important to note that cesarean delivery involves not only somatic pain originating from the abdominal incision but also visceral pain due to the manipulation of the uterus and other internal organs. This dual nature of pain presents a challenge in effectively managing postoperative pain with a single intervention. Although the TAP block has shown promising results in reducing somatic pain, it primarily targets the nerves innervating the abdominal wall and may not adequately address the visceral pain component. Therefore, relying solely on the TAP block for pain management may not provide optimal relief for the comprehensive pain experience after cesarean delivery.

## Conclusions

In the context of multimodal analgesia that excludes intraoperative opioids, the TAP block procedure demonstrates its efficacy in providing analgesia during the early hours after a CS. The impact on relieving pain during movement highlights its potential to support and enhance critical maternal activities. The reduced use of analgesic drugs is a significant advantage, potentially minimizing the acute and long-lasting associated side effects. Additional research is necessary to enhance and optimize the TAP-based multimodal strategy, explore different molecules, and determine appropriate dosages.

## Data Availability

The datasets used and/or analyzed during the current study are available from Massimo Antonio Innamorato on reasonable request.

## Abbreviations

BMI: Body mass index
CS: Cesarean section
dNRS: Dynamic numeric rating scale
INR: International normalized ratio
IO: Internal oblique
NRS: Numeric rating score
rNRS: Resting numeric rating score
NSAID: Non-steroidal anti-inflammatory drug
TA: Transversus abdominis
TAP Block: Transversus abdominis plane
US: Ultrasound
